# An Increase in Vigorous but Not Moderate Physical Activity Makes People Feel They Have Changed Their Behavior

**DOI:** 10.3389/fpsyg.2020.01530

**Published:** 2020-07-16

**Authors:** Hermann Szymczak, Lucas Keller, Luka J. Debbeler, Josianne Kollmann, Nadine C. Lages, Peter M. Gollwitzer, Harald T. Schupp, Britta Renner

**Affiliations:** ^1^ Psychological Assessment and Health Psychology, Department of Psychology, University of Konstanz, Konstanz, Germany; ^2^ Social Psychology and Motivation, Department of Psychology, University of Konstanz, Konstanz, Germany; ^3^ Department of Psychology, New York University, New York, NY, United States; ^4^ General and Biological Psychology, Department of Psychology, University of Konstanz, Konstanz, Germany

**Keywords:** physical activity, health behavior, behavioral goals, change, beliefs

## Abstract

**Objective**: While behavioral recommendations regarding physical activity commonly focus on reaching demanding goals by proposing “thresholds,” little attention has been paid to the question of how much of a behavioral change is needed to make people feel that they have changed. The present research investigated this relation between actual and felt behavior change.

**Design**: Using data from two longitudinal community samples, Study 1 and Study 2 comprised 614 (63% women) and 398 participants (61% women) with a mean age of 40.9 years (*SD* = 13.6) and 42.5 years (*SD* = 13.4), respectively. Using a stage-approach, participants were classified into four groups by asking them at the respective second measurement to indicate whether they had become more physically active since their last participation 6 months ago (“Changers”), they had tried but did not succeed in becoming more physically active (“Attempters”), they were already physically active on a regular basis (“Regular Actives”), or they had not tried to become more physically active (“Non-Attempters”). Physical activity was measured using the International Physical Activity Questionnaire (IPAQ), and fitness level was assessed as physical working capacity (PWC) *via* bicycle ergometry. Mixed ANOVAs including Time and Perceived Change as within and between factors were conducted, followed up by simple effect analyses.

**Results**: Participants stating to have become more active in the past 6 months (Changers) showed a significant increase in vigorous physical activity but not in moderate physical activity, with an average of 6.8 (Study 1) and 10.6 (Study 2) metabolic equivalent value-hours (MET-hours) per week in vigorous activity. Corroborating these findings, objective fitness also significantly increased in the group of Changers. No systematic change in moderate or vigorous physical activity was observed for the three other “non-changer” groups (Regular actives, Attempters, Non-Attempters).

**Conclusion**: The intensity of physical activity is the crucial variable for people’s perception of change in physical activity. Moderate physical activity seems not to be perceived as an effective means for behavior change. It thus might fail to unfold sufficient motivational impact, despite its known positive effects on health.

## Introduction

There is strong evidence for the health benefits of regular physical activity (Ekelund et al., 2016). Research suggests regular physical activity to be an effective primary and secondary prevention measure with beneficial effects on more than 25 medical conditions and premature mortality (Warburton and Bredin, 2018). While the awareness about how important physical activity can be for both health promotion and chronic disease prevention has increased in key stakeholders and end-users, levels of physical activity have not increased markedly and are often below recommended thresholds (Schwartz et al., 2019). The World Health Organization (2018) estimates that such physical inactivity is widespread, affecting 1 out of 4 adults and 4 out of 5 adolescents worldwide. A recent survey in 28 European countries showed that nearly half of the Europeans report that they never exercise or play sports in a given week, and about half do not engage in any moderate or vigorous physical activity (European Commission, 2017).

Increasing physical activity has become a public health priority, with national and international health organizations implementing mass-media campaigns to communicate recommendations detailing the desired “threshold” level of physical activity (Knox et al., 2015; Schwartz et al., 2019). Physical activity guidelines by the World Health Organization (2010) and national health institutions, that is, in the United States (U.S. Department of Health and Human Services, 2018), Canada (Tremblay et al., 2011; Canadian Society for Exercise Physiology, 2019), Australia (Australian Department of Health, 2014a,b), United Kingdom (National Health Service, 2011; U.K. Department of Health, 2011), and Germany (Bundeszentrale für gesundheitliche Aufklärung, 2017), have been harmonized. It is recommended that adults engage in a minimum of 150 min a week of moderate-to-vigorous physical activity (MVPA). Guidelines provide essential information on the minimum level of physical activity needed for health benefits; they are essential for monitoring efforts, planning interventions, and public policy (Knox et al., 2014).

From a psychological perspective, such guidelines as 150 min of MVPA per week qualify as assigned behavioral goals. These can lead to appropriate action as postulated in discrepancy-reduction based models such as control theory (Carver and Scheier, 1998) and goal-setting theory (Locke and Latham, 2013). A high desirability and feasibility of the aspired-to goal positively affect goal strength (also referred to as goal commitment), and high goal commitment is assumed to promote goal attainment. Moreover, the self-regulation approaches to goal setting and goal striving highlight that it matters how people prospectively think about goal setting and goal implementation. For goal setting, it is important that people contrast their desired outcomes with the present hindrances (see the mental contrasting theory by Oettingen, 2012, 2014), and for goal striving it is important that people plan out when, where, and how they want to act in order to reach their goals [see the mindset theory of action phases (Gollwitzer, 1990); the model of if-then planning (Gollwitzer, 1993, 1999; Gollwitzer and Sheeran, 2006); and the health action process approach (Schwarzer, 1992, 1999, 2008, 2011)].

In general, the link between goal commitment on the one hand and actual taking action on the other has been shown to be weak to moderate only (referred to as the “intention-behavior gap”; e.g., Sheeran et al., 2012; Rhodes and Dickau, 2013; Sheeran and Webb, 2016), and this is true even when individuals perceive a need for change (e.g., Bandura, 1997; Schwarzer and Renner, 2000; Nigg et al., 2008; Baldwin and Sala, 2018). As a consequence, theories and research on the self-regulation of goal setting and goal striving have become very popular in recent years (e.g., Gollwitzer and Oettingen, 2012, 2019; Chang et al., 2017; Thürmer et al., 2017; Avishai et al., 2019), as they try to elucidate what kind of strategies people can use to enhance both goal commitment and the translation of goals into action. Importantly, when the desired target behavior is not a one-time action but requires building a habit towards reaching long-term goals and permanent change (e.g., “I want to become a physically active person”), accumulating smaller behavioral steps becomes an important strategy (e.g., “baby steps”; Fogg, 2009a,b, 2019). Hence, recognizing such small behavioral changes (“baby steps”) as meaningful building blocks for long-term sustainable change should help people to arrive at substantial behavior change.

Considering the current physical activity level of many people, the normative “threshold” of 150 min of MVPA per week might represent a rather challenging behavior change goal. Furthermore, Slotterback et al. (2006) coined the phrase “no pain, no gain” to capture the finding that people might erroneously believe that physical activities must be intense to be of benefit. However, the emphasis on normative time and high-intensity goals may actually deter some people from becoming physically active because the difference between these normative recommendations and the actual physical activity level (“reality-norm gap”) is big and may thus be demotivating (Slotterback et al., 2006; Knox et al., 2014; Schwartz et al., 2019). In contrast, small but meaningful changes in behavior which are easily obtainable and associated with repeated incentives may quickly become part of a person’s self-regulation efforts, thereby achieving the desired “ultimate” behavior change. This perspective however calls for research on the personal perception of behavior change. A critical issue concerns the needed amount or intensity of instrumental behaviors that allows people to feel that they have moved towards the desired goal. One hypothesis is that people only notice a behavior change when they substantially increase the intensity of their physical activity in line with the “no pain, no gain” metaphor. Alternatively, people may already recognize comparably small increases (i.e., “baby steps”) in their physical activity as a behavior change, as a meta-analysis could demonstrate health benefits already for low-intensity physical activity (Warburton and Bredin, 2016). Although the question of how much people should change (normative goal or injunctive norm) and to what degree they attained normative goals (objective behavior change) has received considerable attention in research (Shilts et al., 2004; Foster et al., 2009; Wen et al., 2011; Piercy et al., 2018; U.S. Department of Health and Human Services, 2018), the question of how much behavior change is necessary to make people feel that they have changed their behavior (subjective behavior change) has rather been neglected (Zahrt and Crum, 2017).

Accordingly, the main aim of the present study was to shed light on the relation between the felt and actual behavioral change in order to examine whether subjective change is reflected in actual change and which kind of behavior is driving subjective feelings of change. For Study 1, data was taken from the Konstanz Life-Study, a longitudinal multiple-cohort study, including observations from two time points which were 6 months apart. To assess perceived behavior change, participants were asked to indicate if they had been more physically active over the past 6 months, attempted but failed to increase physical activity, were already high in physical activity, or did not attempt to increase physical activity; they were then classified accordingly in four groups of perceived behavior change (Changers, Attempters, Regular Actives, and Non-Attempters, respectively). We furthermore, assessed self-reported physical activity and objective fitness *via* ergometer tests at each time point, in order to find out what amount of change in physical activity corresponds to the perception that one has increased the level of physical activity. In a first step, we examined whether study participants who reported that they increased their physical activity in the past half year (“Changers”) actually showed a significant change in their physical activity level compared to three other groups who did not report a change in their physical activity. Specifically, we hypothesized that “Changers” reported a greater change in their activity behavior as compared to the three other groups. Further analysis analyzed levels of vigorous and moderate physical activity separately. According to the “no pain, no gain” assertion, changes in vigorous physical activity level should be more impactful regarding perceived behavior change than changes in moderate intensity. The reproducibility of findings has become a central issue in the social sciences (Brandt et al., 2014; Simons, 2014; Open Science Collaboration, 2015; Wicherts, 2017). To follow-up the findings from Study 1, we conducted a second study providing a direct replication of the main findings of Study 1. Specifically, data were collected of a new but similar sample and holding all of the research methods and procedures constant (see National Science Foundation, 2018).

## Materials and Methods

### Procedure and Participants for Studies 1 and 2

Data were collected under the Konstanz Life-Study, an ongoing longitudinal multiple-cohort study launched in spring 2012 (e.g., Renner et al., 2012; Sproesser et al., 2015, 2017; Klusmann et al., 2016; Gamp et al., 2018; König et al., 2018; Konstanzer Life Studie, 2019)^1^. The overall goal of the Konstanz Life-Study is to investigate influences on health behaviors, such as physical activity and dietary behavior, across time. Measurements include fasting blood samples, questionnaires, anthropometric measures, as well as cognitive and physical fitness tests. People aged 18 years and older without acute infectious diseases were eligible for participation in the Konstanz Life-Study. For each time point, new participants were recruited *via* flyers, posters, and newspaper articles. Participants who took part in a preceding time point were re-invited *via* email.

In the present analyses, three time points (TP1 to TP3), which were each half a year apart, were included. For Study 1, participants who attended TP1 (spring 2012) and TP2 (autumn 2012) were examined. For Study 2, participants who took part in TP2 (autumn 2012) and attended TP3 (spring 2013) were analyzed. Since the reliability and validity of the International Physical Activity Questionnaire (IPAQ; Craig et al., 2003) has been demonstrated only for adults aged between 18 and 65 years, only these participants were eligible for analysis.

#### Ethics

For data processing and security, a register of processing operations was developed in cooperation with and approved by the Center for Data Protection of the Universities in Baden-Württemberg (ZENDAS) in 2012 and subsequently reviewed by the Commissioner for Data Protection in Baden-Württemberg. All participants gave written informed consent prior to participation. The study adhered to the guidelines of the German Psychological Society and the Declaration of Helsinki and was conducted in compliance with relevant institutional guidelines. The study protocol was approved by the University of Konstanz Ethics Committee.

#### Study 1

In total, 1,321 participants attended TP1, and out of these, 799 participants took also part in TP2 and, thus, were eligible for analysis in Study 1. Of these 799 participants, 155 participants were excluded because of age, 29 due to missing data with regard to the IPAQ or the behavior change question, and 10 due to excessive physical activity values (metabolic equivalent value-hours per week, MET-hours/week, over 200 which corresponds to more than 25 h of vigorous or 50 h of moderate physical activity). Therefore, the data of 605 participants (61.2% female) were included in the analysis for Study 1 (see Table 1). The sample had a mean age of 40.9 years (*SD* = 13.6), had a mean body mass index (BMI) of 24.6 kg/m^2^ (*SD* = 4.0, ranging from 17.5 to 45.2), and had completed on average 15.9 years of education (*SD* = 2.4, ranging from 10 to 20). Compared to the German population (Statistisches Bundesamt, 2017, 2018a), the sample was 3.4 years younger, comprised 10% more females, and had a slightly lower average BMI (the average BMI of the German population is 26 kg/m^2^; Statistisches Bundesamt, 2018b).

**Table 1:** Sample characteristics of Studies 1 and 2.

	Study 1	Study 2
	*M* (*SD*)	*M* (*SD*)
*N*	605	382
Sex (% female)	61%	61%
Age	40.9 (13.6)	42.5 (13.4)
Years of education	15.9 (2.4)	16.2 (2.3)
BMI (kg/m^2^)	24.6 (4.0)	24.4 (3.8)

BMI, body mass index; Age, years of education, and BMI in mean (*SD*).

Eligible (*n* = 605) and non-eligible participants (*n* = 716) did not differ significantly regarding BMI, *t*(1257) = 1.56, *p* = 0.12, or gender, *χ* ^2^(1) = 1.28, *p* = 0.259, *ϕ* = 0.032. However, with a mean age of 46.4 (*SD* = 20.4) and an average education of 15.3 (*SD* = 2.5) years, the non-eligible participants were significantly older, *t*(1272) = −5.65, *p* < 0.001, and reported slightly fewer years of education, *t*(1249) = 4.93, *p* < 0.001, than the eligible participants. However, this is mostly because participants older than 65 had to be excluded because the IPAQ had not been validated for this age group.

#### Study 2

For Study 2, 883 participants attended TP2, and out of these, 587 participants also attended TP3 and, thus, were eligible for analysis. Of these 587 participants, 140 were excluded because of age, 56 due to missing data with regard to the IPAQ or the behavior change question, and 9 due to excessive physical activity (MET-hours/week >200). Therefore, the data for 382 participants (60.7% female) were included in the analysis for Study 2 (see Table 1). The sample had a mean age of 42.5 years (*SD* = 13.4), had a BMI of 24.4 kg/m^2^ (*SD* = 3.8, ranging from 17.5 to 40.8), and had completed on average 16.2 years of education (*SD* = 2.3, ranging from 9 to 20). Compared to the German population (Statistisches Bundesamt, 2017, 2018a) the sample was 1.8 years younger, comprised 10% more females, and had a slightly lower average BMI (Statistisches Bundesamt, 2018b).

Eligible participants (*n* = 382) and non-eligible participants (*n* = 501) did not differ significantly regarding gender, *χ* ^2^(1) = 2.07, *p* = 0.15, and *ϕ* = 0.051. However, they differed in age, BMI, and years of education. The non-eligible participants with a mean age of 52.9 (*SD* = 20.1), an average BMI of 25.2 kg/m^2^ (*SD* = 3.9), and an average education of 15.4 (*SD* = 2.5) years, were older, *t*(814) = −8.14, *p* < 0.001, had a higher BMI, *t*(803) = −2.77, *p* < 0.01, and reported slightly fewer years of education, *t*(801) = 4.87, *p* < 0.001, than the study sample.

### Measurements

#### Physical Activity

Physical activity was assessed using an adapted version of the Short Form of the IPAQ (Craig et al., 2003; The IPAQ Group, 2005; van Poppel et al., 2010; Helmerhorst et al., 2012). Participants reported their physical activity for each of the last 7 days and the following domains: vigorous physical activity, moderate physical activity, and walking. Level of physical activity is calculated as total MET-hours per week which is a unit for the metabolic cost of physical activity and thus an indicator of the intensity of physical activity (U.S. Department of Health and Human Services, 1996; Ainsworth et al., 2000). Based on the IPAQ guidelines (The IPAQ Group, 2005), MET-values of 8, 4, and 3.3 were assigned to vigorous physical activity, moderate physical activity, and walking, respectively. The sum of moderate and vigorous physical activity served as a measure of MVPA.

#### Perceived Change in Physical Activity

Perceived change was assessed at the respective second measurement used in both studies. After reading the item “Since your last participation in the Konstanz Life-Study, have you been more physically active than before?”, participants were asked to choose the one statement they would agree with the most regarding their physical activity behavior: (1) Changers: (“*Yes, I became more physically active*”), (2) Attempters: (“*No, but I tried to become more physically active*”), (3) Non-Attempters: (“*No and I have not (even) tried*”), and (4) Regular Actives: (“*No, because I was already physically active on a regular basis before*”). Each answer represents different stages of intention and behavior (c.f., Klusmann et al., 2016).

#### Objective Fitness

To assess the objective fitness level, physical working capacity (PWC) was assessed *via* bicycle ergometry with pulse monitoring (see also Klusmann et al., 2016). Participants were instructed to try holding up the pedaling rate close to 60 min^−1^. The test started at 25 W and the load was increased by 25 W every 60 s until either pre-determined maximum PWC value (adjusted for age) was reached or participants indicated to be exhausted. The PWC index refers to the physical performance of a person measured in watts at a specific heart rate (here: 130, i.e., PWC 130) divided by body weight (W/kg). The higher the PWC, the better a person’s physical fitness. To be eligible for this test, participants’ blood pressure had to be in the normal range (systole below 150 mmHg and diastole below 100 mmHg). Participants who reported cardiovascular disease/events, lung disease, metabolic disorders, mental disorders with physical exercise counter indicated, epilepsy, multiple sclerosis, current antitumor therapy, major intervention, or surgery within the last 12 months, or other severe chronic or acute diseases were also excluded from this assessment, as were women who were pregnant. Hence, PWC analysis was based on data of 499 and 326 participants in Study 1 and Study 2, respectively.

### Statistical Analysis

Data were analyzed using SPSS Statistics 25.0. For total physical activity and PWC, a mixed 4 between (Perceived Change: Changers vs. Attempters vs. Non-attempters vs. Regular Actives) × 2 within (Time: first vs. second measurement) ANOVA was conducted. For follow-up analyses, *post-hoc* tests with Bonferroni correction for multiple comparisons (*α* = 0.0125) were conducted. To capture effect sizes, Cohen’s d for single mean comparisons is reported (Becker, 1988).

To determine differential effects as a function of the intensity of physical activity, a mixed 4 between (Perceived Change: Changers vs. Attempters vs. Non-Attempters vs. Regular Actives) × 2 within (Time: first vs. second measurement) × 2 within (Intensity of the physical activity: moderate vs. vigorous) ANOVA was conducted. Power analyses revealed that the sample sizes of Studies 1 and 2 were able to detect interaction effects of *f* = 0.13 (*d* = 0.27) and *f* = 0.17 (*d* = 0.33), respectively (*α* = 0.05, power of 0.80). Follow-up analyses included separate ANOVAs for vigorous and moderate physical activity and the calculation of simple effects. In order to test the generalizability of our findings, all analyses for changes between T0 and T1 (i.e., Study 1) were replicated for examining changes between T1 and T2 (i.e., Study 2).

## Results

### Total Physical Activity

The first string of analyses focused on the level of physical activity associated with the subjectively perceived behavior change and whether this relation only held for the group of Changers.

#### Study 1

**Figure 1:**
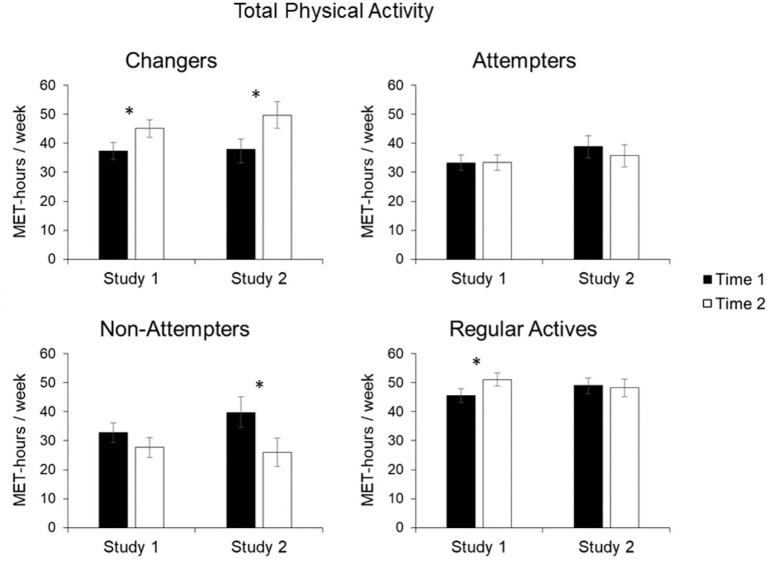
Mean total physical activity in metabolic equivalent value (MET)-hours/week for Changers, Attempters, Non-Attempters, and Regular Actives. Error bars represent standard error of the mean (SE). Statistically significant changes from Time 1 to Time 2 are indicated by asterisks (^✽^indicates *p* < 0.05).

The significant main effect Perceived Change, *F*(3, 601) = 12.51, *p* < 0.001, *η_p_* ^2^ = 0.059, was qualified by the significant interaction of Perceived Change and Time, *F*(3, 601) = 2.74, *p* = 0.042, *η_p_*^2^ = 0.014. As shown in Figure 1, Changers increased their level of physical activity by a value of 7.8 MET-hours per week from the first to the second measurement, *t*(148) = −2.56, *p* < 0.05, *d* = 0.20. No change in physical activity was observed for the Attempters, that is, participants who stated that they unsuccessfully tried to increase physical activity, *t*(157) = −0.03, *p* = 0.973, and the Non-Attempters, that is, participants who have not tried to change, *t*(78) = 1.27, *p* = 0.207. While the Regular Actives also increased their level of activity from first to second measurement, *t*(218) = −2.11, *p* < 0.05, the value of *p* exceeded the pre-determined *α* = 0.0125 to correct for multiple comparisons.

#### Study 2

Overall, findings from Study 2 were similar to those of Study 1. Again, the main effect of Perceived Change, *F*(3, 378) = 5.05, *p* = 0.002, *η_p_* ^2^ = 0.039, was qualified by the significant interaction of Perceived Change and Time, *F*(3, 378) = 4.41, *p* = 0.005, *η_p_* ^2^ = 0.034. Again, the results show that the Changers significantly increased their level of activity. On average, they increased their physical activity by 11.8 MET-hours per week (Figure 1) from the first to the second measurement, *t*(78) = −2.43, *p* < 0.01, *d* = 0.32. Conversely, the group of Attempters and Regular Actives showed no significant change in their physical activity, *t*(96) = 0.71, *p* = 0.481, and *t*(150) = 0.28, *p* = 0.78, respectively. Non-Attempters showed a non-significant decline in their activity, *t*(54) = 2.84, *p* < 0.01, as the value of *p* exceeded the pre-determined *α* = 0.0125 to correct for multiple comparisons.

### Intensity of Physical Activity

In a second step, we added the factor of Intensity to increase the sophistication of our analysis by differentiating between vigorous and moderate physical activity. This allows differentiating effects of the intensity of physical activity on the perception of behavior change.

#### Study 1

Significant two-way interactions of Perceived Change and Intensity, *F*(3, 601) = 11.13, *p* < 0.001, *η_p_* ^2^ = 0.053, and Perceived Change and Time, *F*(3, 601) = 2.74, *p* = 0.042, *η_p_* ^2^ = 0.014, were qualified by a significant three-way interaction of Perceived Change, Intensity, and Time, *F*(3, 601) = 2.68, *p* = 0.046, *η_p_* ^2^ = 0.013. To follow-up on the three-way interaction effect, separate analyses were conducted for vigorous and moderate physical activity.

**Figure 2:**
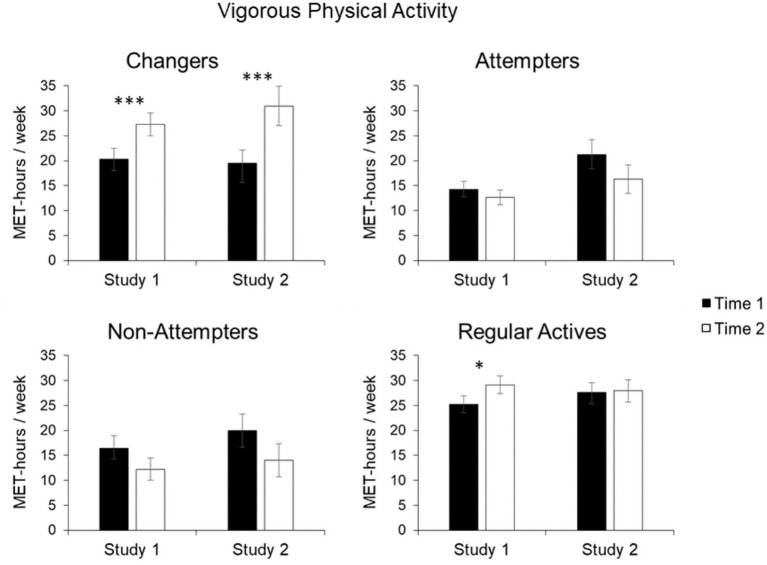
Mean vigorous physical activity in MET-hours/week for Changers, Attempters, Non-Attempters, and Regular Actives. Error bars represent SE of the mean. Statistically significant changes from Time 1 to Time 2 are indicated by asterisks (^✽^indicates *p* < 0.05, ^✽✽✽^indicates *p* < 0.001).

For *vigorous physical activity*, the significant main effect of Perceived Change, *F*(3, 601) = 17.57, *p* < 0.001, *η_p_* ^2^ = 0.081, was qualified by the significant interaction of Perceived Change and Time, *F*(3, 601) = 4.86, *p* = 0.002, *η_p_* ^2^ = 0.024. As shown in Figure 2, Changers had increased their level of vigorous physical activity by 6.9 MET-hours per week from the first to the second measurement, *t*(148) = −3.18, *p* < 0.01, *d* = 0.25. No change in vigorous physical activity could be observed for the Attempters, *t*(157) = 1.20, *p* = 0.234, and the Non-Attempters, *t*(78) = 1.58, *p* = 0.118. Similar to the analysis of the total physical activity, the Regular Actives increased their level of vigorous physical activity from first to second measurement, *t*(218) = −1.93, *p* < 0.05, however, the value of *p* exceeded the pre-determined *α* = 0.0125 to correct for multiple comparisons.

**Figure 3:**
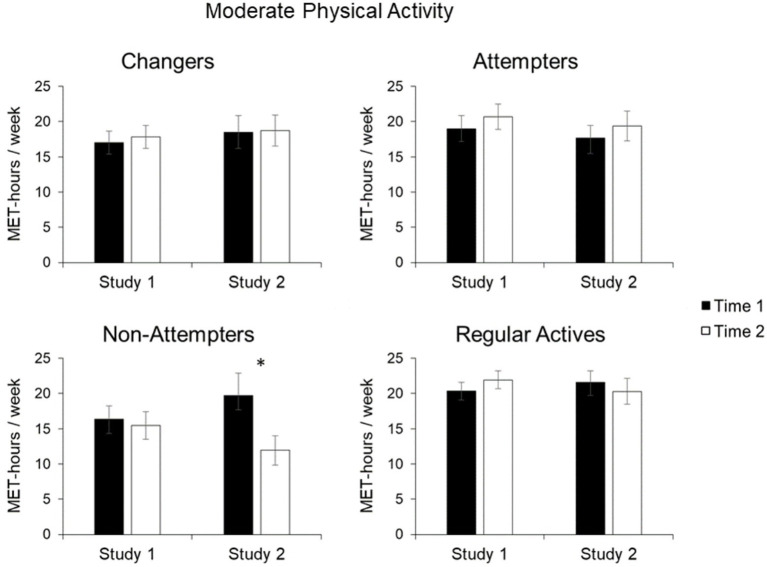
Mean moderate physical activity in MET-hours/week for Changers, Attempters, Non-Attempters, and Regular Actives. Error bars represent SE of the mean. Statistically significant changes from Time 1 to Time 2 are indicated by asterisks (^✽^indicates *p* < 0.05).

A different pattern of findings emerged for *moderate physical activity*. The significant main effect of Perceived Change, *F*(1, 601) = 2.85, *p* = 0.037, *η_p_* ^2^ = 0.014, was not further qualified by the interaction of Perceived Change and Time, *F*(3, 601) = 0.25, *p* = 0.863. Furthermore, exploratory analyses of simple effects showed no significant change in moderate physical activity for any of the four groups between time points (Figure 3), *F*s(1, 601) < 1, *p*s > 0.30.

#### Study 2

The two-way interaction of Perceived Change and Time, *F*(3, 378) = 4.41, *p* = 0.005, *η_p_* ^2^ = 0.034, was qualified by the significant three-way interaction of Perceived Change, Intensity, and Time, *F*(3, 378) = 3.72, *p* = 0.012, *η_p_* ^2^ = 0.029. Accordingly, separate analyses were conducted for vigorous and moderate physical activity.

For *vigorous physical activity*, key findings from Study 1 were replicated. Specifically, the significant main effect of Perceived Change, *F*(3, 378) = 4.92, *p* = 0.002, *η_p_* ^2^ = 0.038, was qualified by the significant interaction of Perceived Change with Time, *F*(3, 378) = 5.24, *p* = 0.001, *η_p_* ^2^ = 0.040 (Figure 2). Noteworthy, the Changers had increased their level of vigorous physical activity on average by 11.5 MET-hours per week from the first to the second measurement, *t*(78) = −3.2, *p* < 0.01, *d* = 0.39. Furthermore, there were no significant effects for the Non-Attempters, *t*(54) = 1.82, *p* = 0.075, as well as the Regular Actives, *t*(150) = −0.15, *p* = 0.882. Similarly, no change in vigorous activity was observed for the Attempters who, if anything, even showed a decline in vigorous physical activity that however was not statistically significant, *t*(96) = 0.143, *p* = 0.157.

For *moderate physical activity*, the interaction of Perceived Change and Time approached significance, *F*(3, 378) = 2.14, *p* = 0.095. Exploratory analyses of simple group effects indicated no change in moderate physical activity for Changers, *t*(78) = −0.1, *p* = 0.921, Attempters, *t*(96) = −0.76, *p* = 0.449, and Regular Actives, *t*(150) = 0.63, *p* = 0.521. Non-Attempters showed a nonsignificant decline in moderate physical activity from first to second measurement, *t*(54) = 3.17, *p* < 0.05, as the value of *p* exceeded the pre-determined *α* = 0.0125 to correct for multiple comparisons.

### Objective Physical Fitness (PWC 130 Test)

To corroborate the findings based on self-reported physical activity behavior, the analyses of the results of the objective fitness test provide additional insights into changes in physical fitness over time.

#### Study 1

Significant main effects of Perceived Change, *F*(3, 495) = 11.14, *p* < 0.001, *η_p_* ^2^ = 0.063, and Time, *F*(1, 495) = 4.32, *p* = 0.038, *η_p_* ^2^ = 0.009, were qualified by a significant interaction of Perceived Change and Time, *F*(3, 495) = 6.64, *p* < 0.001, *η_p_* ^2^ = 0.039. Accordingly, simple effects were calculated for the four groups of perceived change. A significant increase in physical fitness was observed for the Changers from the first (*M* = 1.58, *SD* = 0.45) to the second measurement (*M* = 1.69, *SD* = 0.44), *t*(123) = −4.33, *p* < 0.001, *d* = 0.25. In contrast, no significant effects emerged for the other three groups, *F*s(1, 495) < 2.1, *p*s > 0.15 (Table 2).

**Table 2:** Changes in physical working capacity (PWC) and walking as a function of Perceived Change between Baseline (B) and Follow-up (F).

	Changers		Attempters		Non-Attempters		Regular Actives	
	B	F	B	F	B	F	B	F
	*M* (*SD*)	*M* (*SD*)	*M* (*SD*)	*M* (*SD*)	*M* (*SD*)	*M* (*SD*)	*M* (*SD*)	*M* (*SD*)
Study 1								
PWC	1.58 (0.45)	1.69 (0.44)	1.58 (0.42)	1.60 (0.41)	1.59 (0.46)	1.55 (0.44)	1.82 (0.44)	1.84 (0.49)
Walking	17.0 (19.5)	17.8 (19.8)	19.0 (23.3)	20.7 (22.7)	16.3 (17.1)	15.5 (17.5)	20.3 (18.7)	21.9 (18.8)
Study 2								
PWC	1.68 (0.41)	1.72 (0.42)	1.61 (0.39)	1.61 (0.41)	1.70 (0.52)	1.76 (0.51)	1.81 (0.52)	1.81 (0.48)
Walking	18.5 (21.7)	18.7 (19.9)	17.6 (18.2)	19.4 (20.9)	19.7 (23.4)	11.9 (15.3)	21.6 (19.7)	20.3 (23.0)

Study 1: baseline (B; spring 2012) and follow-up (F; autumn 2012); Study 2: baseline (B; autumn 2012) and follow-up (F; spring 2013).

#### Study 2

In contrast to Study 1, the significant main effect of Perceived Change, *F*(3, 322) = 3.51, *p* = 0.016, *η_p_* ^2^ = 0.032, was not qualified by a significant interaction of Perceived Change with Time, *F*(3, 322) = 1.18, *p* = 0.318.

### Control Analyses

Several control analyses were undertaken to determine whether the observed findings were moderated by gender. No higher-order interaction involving Perceived Change, Time, and Gender was observed for total physical activity in Studies 1 and 2, *F*(3, 597) = 0.33, *p* = 0.804, and *F*(3, 373) = 0.12, *p* = 0.949, respectively. This holds true for objective fitness (PWC) in Study 1, *F*(3, 491) = 0.04, *p* = 0.990, and Study 2, *F*(3, 317) = 0.28, *p* = 0.837. Similarly, the interaction of Perceived Change, Time, Intensity, and Gender was neither significant in Study 1, *F*(3, 597) = 1.35, *p* = 0.26, nor in Study 2, *F*(3, 373) = 0.67, *p* = 0.572. However, commonly observed gender differences in physical activity were replicated (Ransdell et al., 2004; Bauman et al., 2012; Hallal et al., 2012). Men showed higher total physical activity levels than women, *F*(1, 597) = 9.16, *p* < 0.01, *η_p_* ^2^ = 0.015 and *F*(1, 373) = 12.74, *p* < 0.001, *η_p_* ^2^ = 0.033, in Study 1 and Study 2, respectively. Furthermore, the two-way-interaction of Intensity and Gender was significant, *F*(1, 597) = 13.10, *p* < 0.001, *η_p_* ^2^ = 0.021 and *F*(1, 373) = 14.07, *p* < 0.001, *η_p_* ^2^ = 0.036, for Study 1 and Study 2, respectively. This indicates that men were more active in terms of vigorous activity, *F*(1, 597) = 17.64, *p* < 0.001, *η_p_* ^2^ = 0.029 and *F*(1, 373) = 21.38, *p* < 0.001, *η_p_* ^2^ = 0.054, in Study 1 and Study 2, respectively, but not in terms of moderate physical activity, *F*s < 0.8, *p*s > 0.78. In addition, men had higher PWC-scores than women, *F*(1, 491) = 46.83, *p* < 0.001, *η_p_* ^2^ = 0.087 and *F*(1, 317) = 56.50, *p* < 0.001, *η_p_* ^2^ = 0.151, in Studies 1 and 2, respectively.

Further control analyses examined whether the four change groups differed in walking which represents a light-to-moderate-intensive activity (see Table 2). Similar to moderate physical activity, no significant interaction between Perceived Change and Time was found, *F*(3, 601) = 0.41, *p* = 0.749 and *F*(3, 378) = 0.69, *p* = 0.561, for Study 1 and 2, respectively.

## Discussion

Two studies examined individuals’ perceptions of their physical activity to answer the question of how much change is needed for people to feel that they have changed. Toward this end, self-reported physical activity and objective fitness levels were assessed in a group of participants who claimed that their physical activity levels had increased, and these were compared to three control groups: a group of Attempters, a group of Non-Attempters, and a group of Regular Active individuals. The main finding is that participants who stated that they had become more active compared to 6 months ago, did indeed exhibit an overall increase in their physical activity. Importantly, however, further analyses revealed that this is driven by vigorous physical activity, with participants showing an increase of about 52 and 86 min of vigorous activity per week in Study 1 and Study 2, respectively. Combined with the fact that they did not exhibit any changes in moderate activity, this pattern of results suggests that an increase in intensive physical activity is the critical variable for perceiving a change in one’s physical activity.

Perceiving a change in behavior is a key element in the broad array of theories related to behavior change (Gollwitzer, 1990, 2012; Ajzen, 1991; Bandura, 1997; Carver and Scheier, 1998; Schwarzer, 2011; Oettingen, 2012; Locke and Latham, 2013). Perceived change indicates to people that their desired behavior change is feasible, which raises their commitment to actually achieve the desired behavior change and thus strive for it more persistently when hindrances are encountered. The question of how much and what type of behavior change is needed to make people feel that the desired change is feasible accordingly addresses a critical issue of promoting behavior change. The pattern of our findings suggests that people only feel that they have changed when they exhibited a substantial increase in vigorous physical activity; increases in moderate physical activity did not qualify. The group of participants who reported to have changed showed no increase in the level of moderate physical activity, which actually turned out to be at the level of people who reported failing to change. Thus, our findings strongly suggest that people do not take small increases (“baby steps”) in physical activity into account when they judge whether behavior change has occurred with respect to heightening one’s physical activity.

### Vigorous Versus Moderate Physical Activity

Our findings indicate that perceiving a positive change in physical activity is driven by an increase in vigorous rather than moderate physical activity. However, evidence accumulated in recent years suggests that moderately intense physical activity does already have positive effects on health and well-being (Warburton and Bredin, 2016). For instance, Wen et al. (2011) concluded in their prospective cohort study with more than 400,000 individuals that 15 min a day or 90 min a week of moderate physical activity is already sufficient for marked health benefits. Similarly, focusing on people aged 60 years and older, a meta-analysis by Hupin et al. (2015) reveals that a low dose of moderate physical activity (1–499 MET-minutes per week) reduces mortality by 22%. However, the positive contribution of moderate physical activity to a person’s health, as consistently and robustly detected in large cohort studies and meta-analyses, does not seem to be reflected in people’s subjective perceptions of how physically active they are.

This blind spot regarding the health effects associated with moderate physical activity might hinder people to adopt a healthier life style. Usually, recommendations provided by the WHO are based on threshold messages suggesting, for instance, a minimum of 150 min of moderate physical activity or 75 min of vigorous physical activity for a typical week (World Health Organization 2010, 2018). But even below these thresholds, moving from an inactive to a more active state is already associated with positive health effects, according to the epidemiological data. Hopefully, these findings will ultimately lead to the development of health campaigns valuing also moderate and low-volume physical activity.

### Long-Term Success

While many people adopt the goal to increase physical activity, long-term success seems limited. Subjectively perceived behavior change may be an especially critical variable for maintaining behavior change. In the present set of studies, the time interval of data collection was 6 months, an interval often chosen in intervention studies to identify participants successfully maintaining a behavior change (c.f., Rhodes and Pfaeffli, 2010). Health models distinguish between adoption and maintenance of a desired behavior change (e.g., Kwasnicka et al., 2016). In the adoption phase, people are encouraged to gradually increase their physical activity, that is, to take baby steps (Fogg, 2009b), and a discrepancy between behavior and goal is mandatory. However, with increasing time, it becomes harder and harder for people to perceive a meaningful increase in physical activity as they are getting closer and closer to the goal. In other words, the discrepancy between the goal standard and the status-quo becomes smaller and smaller (e.g., Locke and Latham, 2006). In this maintenance phase, it will therefore be important that people are inventive in revising their goals in terms of content (e.g., try out new physical activities such as dancing and mountain climbing) and structure (e.g., set goals that specify not falling back as the desired outcome) so that experiences of success are still possible. In contrast, blind spots regarding low-intensity physical activity may undermine the experience of fruitful changes as will the setting of new but overly challenging goals.

### Goal Standards and Monitoring Progress

People’s subjective perceptions of success or failure of a change in behavior is crucial for self-monitoring progress towards goals. In one of the few studies taking self-perceived change into account, Bélanger et al. (2011) identified participants who have changed by the researchers’ criteria (actual change) but did not perceive themselves to have changed (subjectively perceived change). However, *how much* change would have been necessary for the participants to actually perceive change was not explored. Furthermore, Klusmann et al. (2016) used perceived change to distinguish between successful and unsuccessful intenders; still, the authors left the question unanswered of how much actual change (if any) had occurred for those who perceived to have changed. Thus, our finding that perceived change is related to an increase in vigorous physical activity adds relevant information to understanding the processes underlying people’s monitoring of their goal progress.

The fact that people ignore the potential beneficial effects of moderate physical activity may be based on the difficulties associated with taking notice of low volume physical activity. Mobile technologies such as smartphones, mobile body monitoring systems (e.g., movisense®, Actigraph®), and self-tracking tools (e.g., Fitbit®, Apple Watch®^2^) can help people to monitor goal progress with respect to small-scale changes in physical activity. Thus, it is comforting to see that innovative new means are developed that facilitate the monitoring of minor changes in physical activity, which as the present studies suggest would otherwise go by unnoticed (see Crum and Langer, 2007).

Perceiving a change in behavior is construed with respect to standards regarding the desired level of physical activity. Intervention programs usually define the target behavior explicitly, and the various aspects involved in effective goal setting are considered in health behavior change interventions (Strecher et al., 1995). Beyond structured interventions and fitness programs, people intending to increase physical activity can also benefit from social norms and/or the dissemination of fitness recommendations provided in mass media, books, and magazines. While the present research has not been designed to reveal the relationship of perceived change and the normative standards participants adhere to, it is important to note that the level of change shown by participants was substantial, going far beyond current recommendations from major organizations. Accordingly, future research is needed probing not only into perceived behavior change but also into considering how normative standards affect an increase in physical activity.

### Limitations

The present studies are not without limitations. The focus in the present studies was on adults aged between 18 and 65 years because our measure of physical activity (i.e., IPAQ) is validated for this age group. However, when including all of the older participants, findings were highly similar to the reported results for adults between 18 and 65 years of age. While sample size was too small to focus on older participants specifically, it would be highly interesting to explore in future research whether older people’s perception of behavior change is more sensitive than that of younger people (a hypothesis that is in line with a more sensitive adjustment of perceived risk across the lifespan; Renner et al., 2007).

Furthermore, the Konstanz Life-Study consists of a community sample volunteering to participate. Within this research paradigm, it is not feasible to examine the correspondence of change in physical activity with the perceived behavior change as a function of baseline physical activity. Thus, expanding on the current findings, future research is needed to address the relationship of actual change in physical activity with perceived behavior change as a function of the physical activity level at baseline (low, medium, and high). Furthermore, reflecting public health concerns (World Health Organization, 2018), the present research focused on perceived increase in physical activity. However, it would be interesting to also consider the issue of perceiving a decrease in physical activity, determining possible differences in the amount of change needed for a perceived decrease versus increase in behavior.

A further limitation of the present studies is that physical activity levels were assessed *via* self-report rather than objectively measured using mobile tracking. As a proxy, we assessed PWC by using a bicycle ergometer test. Corroborating self-report data, we observed an increase in PWC in the group of participants who had been classified as Changers (i.e., people who perceived a change) in Study 1. Noteworthy, this group was the only of the four groups showing a significant change in PWC. However, data from the second study showed no such difference between groups, possibly reflecting the reduced power of the smaller sample of Study 2. Nevertheless, while acknowledging limitations of the PWC analysis the objective fitness data did substantiate our findings regarding perceived change in physical activity.

## Conclusion

Some of our research participants felt that they succeeded in increasing their physical activity level while others did not. The present research determined what kind of actual behavior change was needed that people felt that they changed their behavior. Findings revealed that an increase in high-intensity physical activity is the critical variable for perceiving a positive change in physical activity. This might lead people to reduce their engagement in moderate intensity physical activity. We also observed that the duration of vigorous activity our participants engaged in was substantial in comparison to current health recommendations of international and national health organizations. The present findings thus speak for a change in current health recommendations. Not only vigorous but also moderate physical activity should by highlighted, given that extensive empirical research has shown that moderate physical activity does have enormous positive health consequences as well.

## Abbreviations

ANOVA, Analysis of variance; BMI, Body mass index; IPAQ, International Physical Activity Questionnaire; MET, Metabolic equivalent value; mm Hg, millimeters of mercury; MVPA, Moderate to vigorous physical activity; PWC, Physical working capacity; WHO, World Health Organization.

## Data Availability Statement

The datasets generated for this study are available on request to the corresponding author.

## Ethics Statement

The studies involving human participants were reviewed and approved by the local ethical review board (University of Konstanz) approved the study protocol. The patients/participants provided their written informed consent to participate in this study.

## Author Contributions

BR designed the study. HTS and BR coordinated the research. BR and HS conceived the analyses. HS and HTS drafted the manuscript. HTS and HS analyzed the data. BR, HTS, and HS interpreted the data. LK and PG made substantial contributions to the design of the work. LK, LD, JK, NL, and PG revised the manuscript substantially. All authors contributed to the article and approved the submitted version.

### Conflict of Interest

The authors declare that the research was conducted in the absence of any commercial or financial relationships that could be construed as a potential conflict of interest.

## Footnotes

^1^www.uni-konstanz.de/life-studie

^2^https://quantifiedself.com
